# A reassessment of the genus *Oligoneuriopsis* Crass, 1947 (Ephemeroptera, Oligoneuriidae, Oligoneuriellini)

**DOI:** 10.3897/zookeys.985.56649

**Published:** 2020-11-05

**Authors:** Helen M. Barber-James, Sonia Zrelli, Zohar Yanai, Michel Sartori

**Affiliations:** 1 Department of Freshwater Invertebrates, Albany Museum, Somerset Street, Makhanda (Grahamstown), 6139, South Africa; 2 Department of Zoology and Entomology, Rhodes University, P.O. Box 94, Makhanda (Grahamstown), 6140, South Africa; 3 Unit of Hydrobiology, Laboratory of Environmental Biomonitoring, Faculty of sciences of Bizerta, 7021, Jarzouna, Tunisia; 4 Musée cantonal de zoologie, Palais de Rumine, Place de la Riponne 6, CH-1014, Lausanne, Switzerland; 5 Department of Ecology and Evolution, Biophore, University of Lausanne, CH-1015, Lausanne, Switzerland; 6 School of Zoology, Tel Aviv University, Tel Aviv 6997801, Israel; 7 The Steinhardt Museum of Natural History, Tel Aviv University, Tel Aviv 6997801, Israel

**Keywords:** Barcoding, generic concept, imaginal description, mayfly, neotype designation, new combination, nymphal description

## Abstract

The distinction between the two closely related genera *Oligoneuriella* Ulmer, 1924 and *Oligoneuriopsis* Crass, 1947 has been much debated. First described from South Africa, *Oligoneuriopsis* seemed to be a clearly defined genus. However, as the known distribution of the genus widened and knowledge on it expanded, species delimitation based on morphology became less clear due to overlap in several apparently defining morphological characters, especially in the nymphs. This work attempts to reassess *Oligoneuriopsis* morphology in the context of all currently known species. The type species, *Oligoneuriopsis lawrencei* Crass, 1947 is redescribed at the imaginal and nymphal stages and a neotype is designated. The putative nymph of *Oligoneuriopsis dobbsi* (Eaton, 1912) is described based on material collected around Mt Elgon (Kenya). The adults of *Oligoneuriella orontensis* Koch, 1980 are described for the first time and the species is transferred to the genus *Oligoneuriopsis* (*Oligoneuriopsis orontensis***comb. nov.**). Egg structure is also described for the first time for the species *Oligoneuriopsis skhounate* and *O. orontensis*. Some biogeographical considerations are also given. It is likely that more species will still be discovered, especially in Africa.

## Introduction

Up to the middle of the 20^th^ century, the only oligoneuriid genus widespread in the Afrotropical realm was *Elassoneuria* Eaton, 1881. In 1947, Crass described the genus *Oligoneuriopsis* to accommodate a new species, *O. lawrencei*, from the region then known as Natal (now KwaZulu-Natal), South Africa. Crass’ genus differs from *Elassoneuria*, the other genus occurring in sub-Saharan Africa, in wing venation differences in the adult stage, male genitalia features, and by the shape of the head in the nymphal stage (Crass 1947). He noted that his new genus somewhat resembles the Palaearctic genus *Oligoneuriella* Ulmer, 1924 (sub. nom. *Oligoneuria* Pictet, 1843), both in nymphal and adult stages. Subsequently, Demoulin (1952) produced a key to identify the adults of Oligoneuriidae, and gave as discriminating characters 4-segmented gonostyli and dissimilar tarsal claws in *Oligoneuriopsis* vs. 3-segmented gonostyli and both tarsal claws obtuse in *Oligoneuriella* (not to be confused with *Elassoneuria*, which also has 3-segmented gonostyli and both tarsal claws obtuse). A nymph of *Oligoneuriopsis* sp. was later recorded from West Africa (Verrier 1958), but according to Demoulin (1970), this specimen belongs to the genus *Elassoneuria*. A second species of *Oligoneuriopsis* was recognized by Kimmins for a species originally described by Eaton as *Oligoneuria dobbsi* from British East Africa (now Kenya) (Eaton 1912) in which male gonostyli are 4-segmented (Kimmins 1960). Two new species were later described from South Africa, based on nymphs collected in the eastern Transvaal (now Mpumalanga) and in KwaZulu-Natal (Agnew 1973). A major advancement was the description of a new species, *Oligoneuriopsis skhounate*, from both nymphal and adult stages from Morocco, extending the known distribution of the genus all the way to North Africa (Dakki and Giudicelli 1980). The recent description of the nymph of *Oligoneuriopsis villosus* from Iran (Sroka et al. 2019) extends the distribution of this genus north-eastwards, to include Southwest Asia as well.

Dakki and Giudicelli (1980) compared *O. skhounate* to *O. lawrencei* and *O. dobbsi*, and proposed additional characters to distinguish the nymphs of *Oligoneuriopsis* from those of *Oligoneuriella*:

- posterolateral abdominal spines on segments II to VII long and going beyond the posterior margin of the tergites in Oligoneuriella compared to Oligoneuriopsis where these spines are shorter, and
- gill lamellae longer than the half length of the corresponding tergite in Oligoneuriopsis and shorter than the half-length in Oligoneuriella.

Neither of these characters holds true for separating the nymphs of these two genera, as in the three South African *Oligoneuriopsis* species, the posterolateral abdominal spines on segments II to VII extend beyond the margin of the tergites, and the gill lamellae are shorter in several species.

The first record of *O. skhounate* in the Iberian Peninsula was published by Gonzalez del Tanago and Garcia de Jalon (1983). In the same year, *O. skhounate* was reported from Algeria (Soldán and Thomas 1983), but the authors transferred it to the genus *Oligoneuriella* without much discussion, saying that “This species actually belongs to the genus *Oligoneuriella* Ulmer as apparent from wing venation and nymphal morphology (Crass 1947; Agnew 1973)”. The species was then recorded from Tunisia (Boumaiza and Thomas 1986), and later on again from Algeria (Gagneur and Thomas 1988) but placed in the genus *Oligoneuriopsis* without generic discussion.

Due to the lack of clear distinctions between *Oligoneuriella* and *Oligoneuriopsis* nymphs, the generic concept relies mainly on male adult differences. Characters used to discriminate between the adults were proposed by Demoulin (1952). Of particular use is the number of segments composing the gonostyli, i.e., three in *Oligoneuriella* (one long proximal and two smaller apical) vs. four in *Oligoneuriopsis* (one long proximal and three smaller apical). Unfortunately, although this character is generally reliable and constant at a generic level in most mayfly families, it appears to be subject to some variation in Oligoneuriidae, as already mentioned by Eaton (1883–1888) and illustrated by Grandi (1947) for the species *Oligoneuriella rhenana* where sometimes a fourth terminal segment is present; the same has been reported by Gillies (1974) for different species of *Elassoneuria* and by Elouard (pers. comm.) for some species of *Madeconeuria*.

Another problem concerns the original description of *Oligoneuriopsis lawrencei* by Crass (1947). The gills present on the first abdominal segment supposedly lacked a lamella, being composed only of a bunch of fibrillae, the most important character fixed by the author to distinguish his new genus from *Oligoneuriella*. When describing *Oligoneuriopsis jessicae* and *Oligoneuriopsis elisabethae*, Agnew (1973) stated that first gill of both species did possess a lamella but did not mention anything about Crass’ species. It was only later (Agnew 1980) that he formally confirmed that Crass’ description was erroneous and that *O. lawrencei* also possessed a lamella on the ventral first gill. A second problem arose when looking at the single illustration we have of the male genitalia of *O. lawrencei* (see Crass 1947: fig. 4a), where the author has drawn penis lobes with lateral sclerite thin and hardly enlarged at the apex (proximal part of the sclerite is unfortunately not drawn), comparable to those found on different species of *Oligoneuriella* (Sowa 1973; Soldán and Landa 1977; Dakki and Giudicelli 1980; Alba-Tercedor 1983; Mol 1984). This character is quite different from what can be seen in *O. dobbsi* and *O. skhounate* where the lateral sclerite is club-shaped and enlarged at apex (Kimmins 1960; Dakki and Giudicelli 1980). As stated by Kluge (2004), the shape of the proximal part of the penis sclerite is apically pointed or bifid in *Oligoneuriopsis dobbsi* and *O. skhounate*, whereas it is saddle-shaped in all *Oligoneuriella* species (see Dakki and Giudicelli 1980 for a good comparison).

Another character to distinguish nymphs of *Oligoneuriella* and *Oligoneuriopsis* has been briefly mentioned by Demoulin (1970) and studied in more detail by Agnew (1980). In the three *Oligoneuriopsis* species from South Africa, Agnew noticed that sternites II-IV or II-V possess a well-developed tuft of long and thin setae in medioposterior position, whereas at least some *Oligoneuriella* species possess instead a tuft of short and stout setae (Sowa 1973). In a recent phylogeny based on a combination of morphology (using *O. skhounate* from Spain), and genetics (using *O. lawrencei* from South Africa), Massariol et al. (2019) indicate that *Oligoneuriopsis* clusters separately from but closely to *Oligoneuriella* within the Oligoneuriidae, and proposed to group these two genera in a new tribe, the Oligoneuriellini.

Finally, a recent paper by Sroka et al. (2019) deals with the description of the nymph of a new *Oligoneuriopsis* species from Iran. The authors propose new diagnostic characters to distinguish their nymphs from those of *Oligoneuriella*. They validate the discriminant character constituted by the length of medioposterior setae on the proximal sternites, which are always long in *Oligoneuriopsis* and short in *Oligoneuriella*. Two other differences are also proposed, namely a row of setae along the entire length of posterior margin of femora and outer margin of tibiae on middle and hind legs found in *Oligoneuriopsis* only.

The aim of the present paper is to investigate and confirm the generic status in reviewing all species presently known. This will also allow us to reassess the generic position of the species *Oligoneuriella orontensis* Koch, 1980, based on the rich material collected from Israel (see Yanai et al. 2020).

## Materials and methods

Material was examined from South Africa, Kenya, Israel, Algeria, Tunisia, Morocco, Iran and the Iberian Peninsula. Material includes nymphs (indicated as N), female subimagoes (s♀), male subimagoes (s♂), female imagoes (♀) and male imagoes (♂). All studied specimens belong to the following collections: Albany Museum, Grahamstown, South Africa (**AMGS**), Museum of Zoology, Lausanne, Switzerland (**MZL**), Laboratory of Environment Biomonitoring, University of Bizerta, Tunisia (**LBE**), and Steinhardt Museum of Natural History, Tel Aviv University, Israel (**SMNH**).

It must be mentioned that all type specimens of *Oligoneuriopsis* from South Africa described by Crass and Agnew are certainly lost (McCafferty and de Moor 1995).

For genetic analysis we used the barcoding genetic marker cytochrome *c* oxidase subunit 1 (COI). We considered available sequences of three *Oligoneuriopsis* species (*O. lawrencei*, *O. orontensis*, and *O. villosus*), to which we added newly obtained sequences of fresh specimens of *O. dobbsi* and *O. skhounate*, thus representing the great majority of the described species in the genus. We used a non-destructive method for DNA extraction, i.e., specimens were incubated overnight soaked at proteinase K to allow DNA extraction without destroying the specimen. Amplification was done using the commonly used primers HCO2198 and LCO1490 (Folmer et al. 1994), with optimal PCR conditions of initial denaturation at 95 °C for 5 min; 38 cycles of 95 °C denaturation for 40 sec, 50 °C annealing for 40 sec, and 72 °C extension for 40 sec; and final stage of 72 °C denaturation for 7 min. To clarify the generic position of *O. orontensis* and as outgroups, we also used representatives of species of the closely related genus *Oligoneuriella* and of other sibling-genera in the family (Table 1). For these taxa we retrieved mitochondrial COI sequences from NCBI GenBank (https://www.ncbi.nlm.nih.gov/genbank/) and from barcode of life database (http://www.boldsystems.org/); accession numbers are given in Table 1.

**Table 1.** Private identifiers of COI sequences used for the genetic analysis (GenBank accession numbers and BOLD process IDs).

Species	Database	Private identifiers
*Oligoneuriopsis lawrencei*	GenBank	MG516468
*Oligoneuriopsis villosus*	BOLD	EPHIR007-19, EPHIR008-19, EPHIR009-19, EPHIR010-19, EPHIR011-19
*Oligoneuriopsis orontensis*	GenBank	MN958842, MN958843, MN958844
*Oligoneuriopsis dobbsi*	GenBank	MT784191
*Oligoneuriopsis skhounate*	GenBank	MT784188, MT784189, MT784190
*Oligoneuriella tuberculata*	BOLD	EPHIR005-19
*Oligoneuriella rhenana*	GenBank	KY262260
*Oligoneuriella pallida*	GenBank	KU609047
*Oligoneuriella tskhomelidzei*	BOLD	EPHIR014-19
*Oligoneuriella bicaudata*	BOLD	BMIKU054-09
*Oligoneuria amazonica*	GenBank	KT201514
*Homoeoneuria watu*	GenBank	MG516463
*Lachlania alcidesi*	GenBank	KU609050
*Madeconeuria* sp.	GenBank	MG516465

We aligned and reconstructed the sequences in MEGA X v.10.0.5 (Kumar et al. 2018). We conducted a Maximum Likelihood (ML) analysis in RAxML v.8 (Stamatakis 2014) as implemented in raxmlGUI v.2.0 beta (Edler et al. 2019); ML analysis was performed with the GTR+G model of sequence evolution (as selected by jModelTest v.2.1.7; Darriba et al. 2012) and 100 replicates. Each inference was initiated with a random starting tree and nodal support was assessed with 1000 bootstrap pseudoreplicates. We conducted the Bayesian Inference (BI) analysis with MrBayes v.3.2.7a (Ronquist et al. 2012); BI analysis was performed with two simultaneous runs, each with four chains (three heated, one cold), for 2*10^6^ generations with sampling every 200 generations. We monitored the standard deviation of the split frequencies between the two runs and the Potential Scale Reduction Factor (PSRF) diagnostic. The first 25% of the trees were discarded as burn-in.

## Results

**Figure 1.**
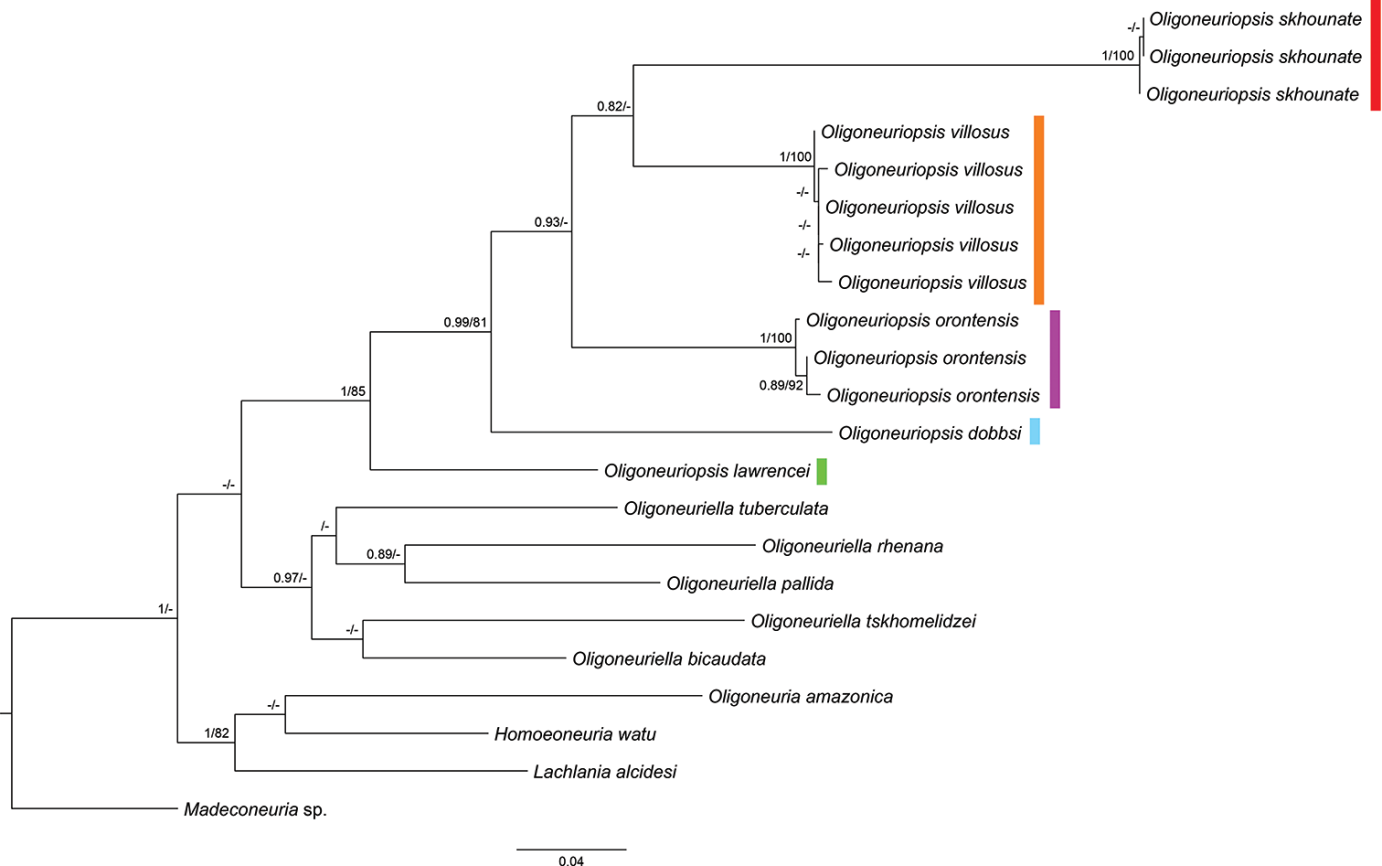
A phylogenetic reconstruction based on Maximum Likelihood and Bayesian Inference analyses of sequences of the mitochondrial COI gene, presented on ML tree. BI posterior probabilities above 0.85 and ML bootstrap values above 70% are indicated next to the nodes.

Figure 1 represents the phylogenetic reconstruction based on Maximum Likelihood and Bayesian Inference analyses of sequences of the mitochondrial COI gene, presented on ML tree. All *Oligoneuriopsis* species are recovered as a monophyletic clade, as are also the *Oligoneuriella* species; the former clade includes *O. orontensis*, thus supports our claim regarding its generic position (see below). Each *Oligoneuriopsis* species presents low intraspecific divergence and high interspecific values, confirming that they are all well-defined genetically. Relationships between *O. orontensis*, *O. villosus* and *O. skhounate* are not clear (BI < 0.85), but both ML bootstrap values and BI indicate that *O. lawrencei* (South Africa) is the sister species of all other *Oligoneuriopsis* species, followed by *O. dobbsi* (Kenya).

## Oligoneuriopsis lawrencei

Taxon classificationAnimaliaEphemeropteraOligoneuriidae

Crass, 1947

BBCCDF44-6EE9-5FB4-8339-751CFB7FB531

**Figure 2.**
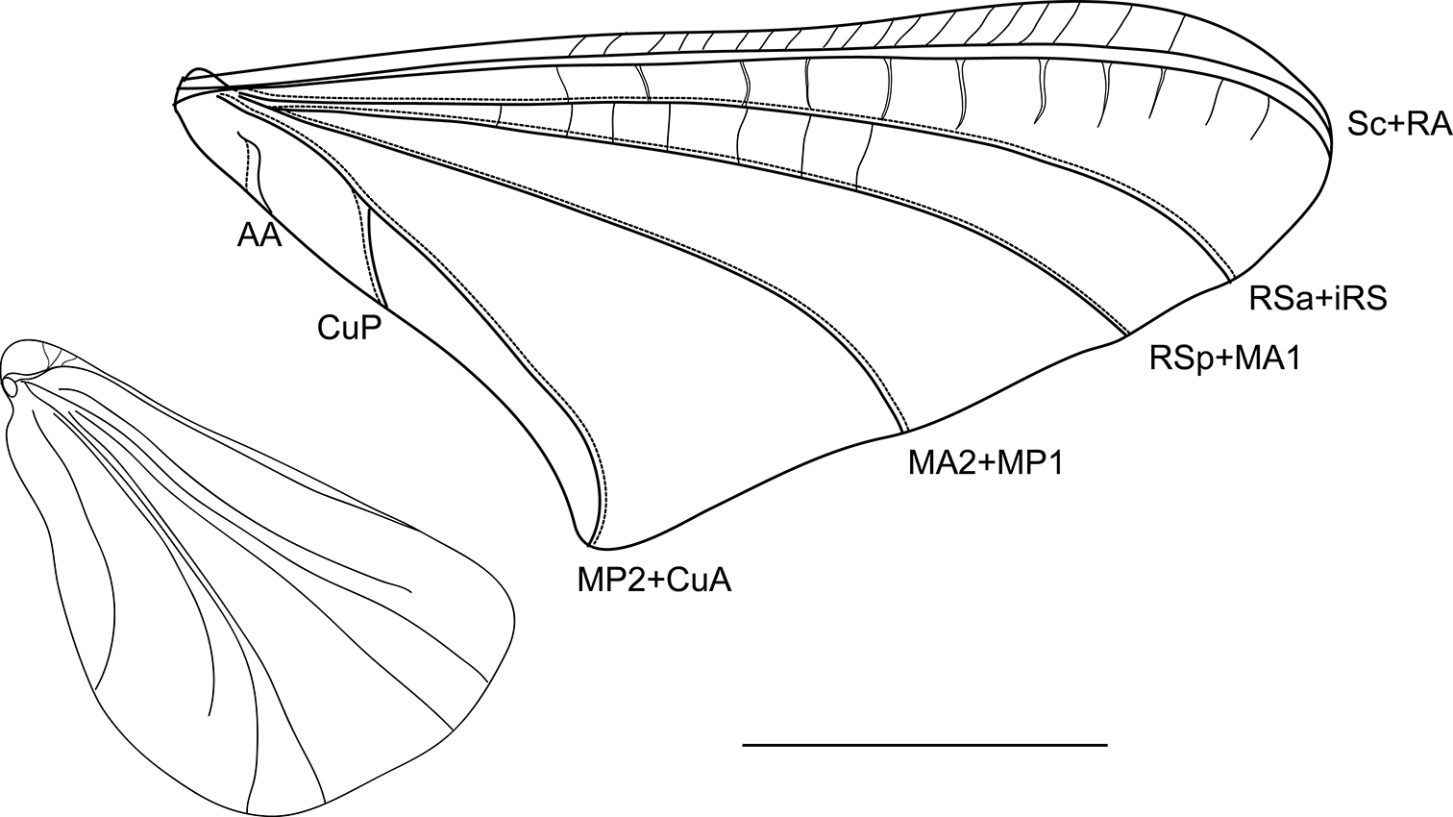
*Oligoneuriopsis lawrencei*, fore- and hind wings. Scale bar 0.5 mm.

**Figure 3.**
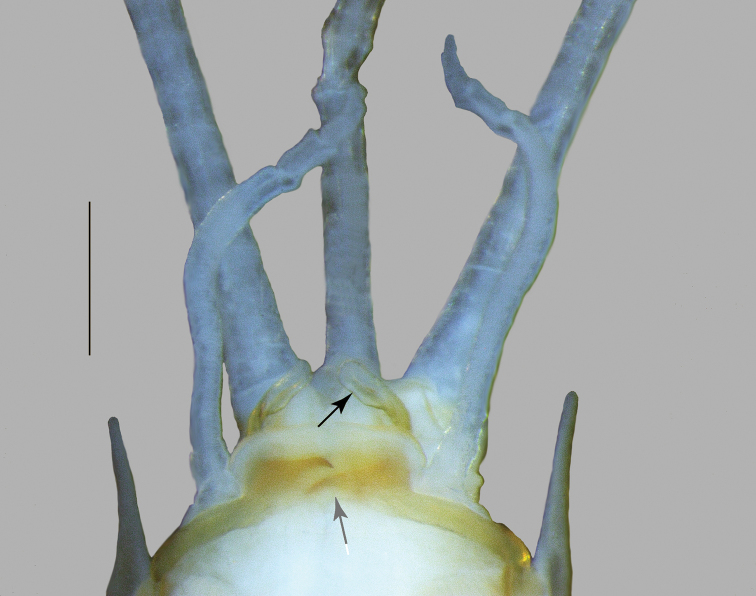
*Oligoneuriopsis lawrencei*, ventral view of lower abdomen of adult male, showing lateral extensions of segment IX, gonostyli and penes. Black arrow: Apex of the lateral longitudinal lobe of penis. Grey arrow: Proximal process of penis. Scale bar 0.5 mm.

**Figure 4.**
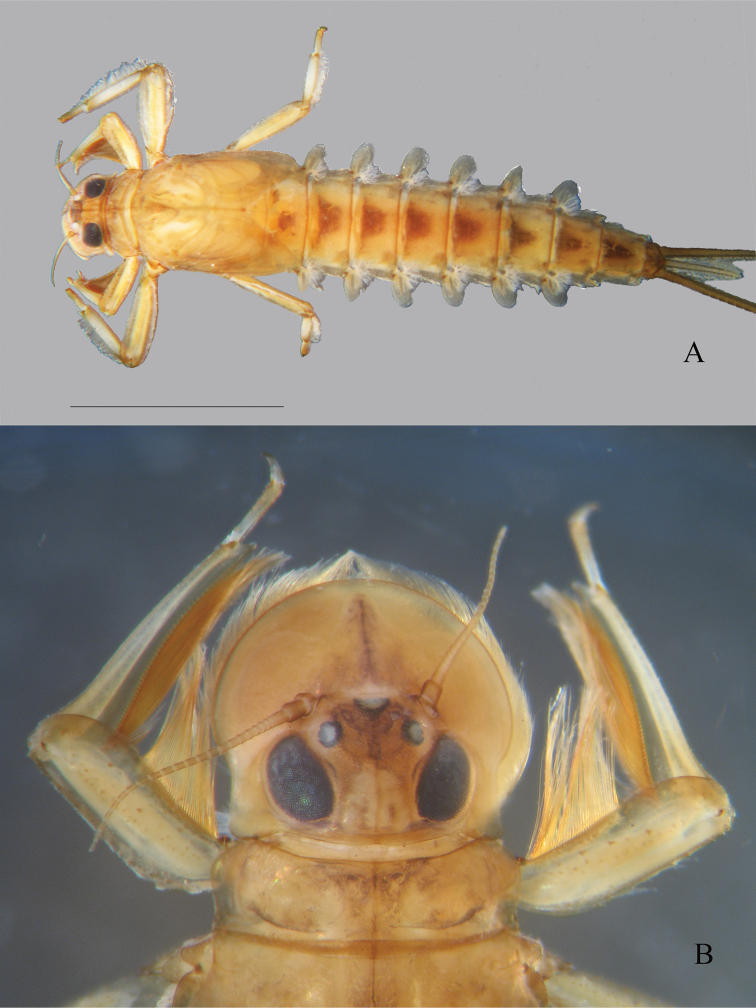
*Oligoneuriopsis lawrencei***A** whole nymph, dorsal view **B** dorsal view of head. Scale bar: 5 mm (**A**).

Figures 2 , 3 , 4 , 14D , 15D

Oligoneuriopsis lawrencei Crass, 1947: 53, figs 3, 4.

### Material examined.

***Neotype***: South Africa • 1N; Eastern Cape Province, Tributary of Tyume River, below Tor Doone, Hogsback; 32.5778°S, 26.9347°E; alt. 1445 m a.s.l.; 29 Feb. 1992; F.C. de Moor leg.; AMGS; GEN 1097A; H.M. Barber-James design., 2020. This specimen was chosen because it comes from one of Crass (1947)’s original localities.

***Other material***: South Africa • 1N; Eastern Cape Province, Tsitsa River, at “The Falls”; 31.0214°S, 28.4819°E; alt. 1140 m a.s.l.; 26 Mar. 1991; F.C. de Moor & H.M. Barber leg.; AMGS; ECR 92AE • 6N; same locality; 28 Mar. 1993; F.C. de Moor & H.M. Barber-James & K. Martens leg.; AMGS; ECR 134A • 12N; Eastern Cape Province, Nqancule River, at Waterval; 31.3672°S, 28.2167°E; alt. 1220 m a.s.l.; 24 Mar. 1991; F.C. de Moor & H.M. Barber leg.; AMGS; ECR 86A • 6N; Eastern Cape Province, Nqancule River, at Albany; 31.3486°S, 28.2153°E; alt. 1240 m a.s.l.; 5 Mar. 1991; F.C. de Moor & H.M. Barber leg.; AMGS; ECR 88A • 1N; Eastern Cape Province, Kettle Spout waterfall, Tyume River tributary, Hogsback; 32.5500°S, 26.9500°E; alt. 1835 m a.sl.; 19 May 2007; F.C. de Moor and N. Phaliso leg.; AMGS; GEN 1845A • 3♀, 4♂, 1N; KwaZulu-Natal, Klein Mooi River, at Durleigh Farm; 29.2283°S, 29.8997°E; alt. 1392 m a.s.l.; 15 Mar. 1995; C. Dickens; AMGS; MOI 29BS • 3♀ 4♂; same locality; 3 Apr. 1995; F.C. de Moor leg; AMGS; MOI 35B.

### Male imago.

Lengths. Body: up to 14.8 mm; forewing: up to 14.9 mm; cerci: up to 17.0 mm; caudal filament: up to 12.8 mm.

Vertex light brown, frontoclypeus pale cream, broadly rounded apically, compound eyes black, base of ocelli black, ocelli whitish, antennae with scape and pedicel pale cream, first segment and flagellum light brown. Pronotum light brown, margins suffused with dark brown pigmentation. Pterothorax light brown, with pale cream-coloured unsclerotized line between meso and metanotal plate. Mesoscutellar filaments present. Forelegs shorter than mid or hind legs, with outer margin of femora, tibiae and tarsi dark brown, otherwise uniform pale brown colour; mid- and hindlegs cream to light brown, no distinct markings. All three pairs of legs appear to be functional. Tarsal claws paired, blunt. Wings (Fig. 2), when folded, light brown, almost whitish when unfolded. Forewing typical of the genus, with five groups of veins: Sc+RA, RSa+iRS, RSp+MA1, MA2+MP1, and a forked MP2+CuA – CuP vein. Subcostal field with numerous transversal veins, those issued from RA not reaching iRS in the distal forth of the length, those between iRS and MA1 only present in the proximal half.

Abdominal segments uniformly creamish, without distinct patterns, except tergites VII, IX, and X light brown; lateral margins with spine-shaped extensions from segment III to IX, of increasing length towards the posterior. Gonostyli whitish to grey, cerci whitish. Gonostyli 4-segmented, the basal one ca. 3 × the length of segments 2 to 4 combined. Penis lobes almost triangular, with characteristic sclerotized proximal process ending in a simple projection; apex of the lateral longitudinal lobe of penis in a small club-shaped sclerite (Fig. 3). Cerci with whorls of long setae at each junction (not figured).

### Female subimago.

Lengths. Body: up to 17 mm; forewing: up to 18.8 mm; cerci: up to 7 mm; caudal filament: up to 4.5 mm. Colouration as in the male; tibiae and tarsi of all legs appear to be functional. Cerci light to medium brown. Posterior margin of sternite IX deeply concave and rounded.

### Nymph.

Lengths. Body up to 15 mm and 18.5 mm in male and female nymphs respectively; cerci (and caudal filament) up to 9.2 mm (4.0 mm) and 10.1 mm (3.5 mm) in male and female nymphs respectively. General colouration light to medium yellow-brown (Fig. 4A), with dark brown dorso-medial markings, better developed in mature male nymphs than in immatures or females. Head (Fig. 4B) medium brown, with maculation between the compound eyes. Ventrally, head a uniform pale cream colour. Gills at base of maxillae forming a “beard” ventrally at base of head, of similar colour to head in Hogsback specimens, orientated in one plane, parallel to length of body. Pro and mesonotum medium brown, with pale cream-coloured maculae. Legs light brown, femoro-tibial articulation darker, setae of forelegs noticeably darker brown than the legs. Femur and tibia of foreleg shorter than those of mid or hind leg, in all cases, femora and tibiae subequal in length. Setae on the outer margin of mid and hind femora well developed, slightly decreasing in size and reaching the apex (Fig. 14D). Tibiae and tarsi with long, even fringe of setae along entire dorsal margin, interspersed with occasional short spine-like setae. Abdominal tergites uniformly medium brown, each with darker brown marking medially; sternites uniform pale brown, with no markings. Dense patch of posteriorly orientated setae ventromedially on abdominal sternites II–IV, much reduced patch on segments V, further reduced on VI. Gills II–VII almost subequal in size, gill I smaller. On all gills except for gill I, fibrillae shorter than lamella length. Lamella of gill I less than half the length of the fibrillar portion. Lamellae II–VII with long and thin setae on their distal inner margin (Fig. 15D). Posterolateral spines of the abdomen increasing in size posteriorly. Whole nymph (dorsal aspect) and gills as illustrated by Agnew (1980). Cerci uniformly medium brown, caudal filament paler brown.

### Intraspecific variation.

Specimens from KwaZulu-Natal are darker brown in colour than specimens with more southerly distribution, and first three abdominal segments darker brown in colour dorsally than remaining segments; faint paired median spots visible on the last five abdominal tergites in some KwaZulu-Natal nymphs. The head also possesses a dark brown marking present on the frons between the ocelli, and maxillary gills are much paler in colour relative to head capsule in KwaZulu-Natal specimens.

### Affinities.

Winged stages were collected from the same locality as the nymphs in one instance, allowing association of the life stages. Imagos were seldom flying at the times of collection. Nymphs of *O. lawrencei* have a broadly rounded fronto-clypeal region, which easily distinguishes them from *O. jessicae* and *O. elisabethae*, both of which are more pointed in shape. Head very slightly carinate, less so than in *O. elisabethae*. Note that this is unlike the strong carination seen in *Elassoneuria*.

### Habitat preference.

Found under large boulders (400–500 mm diameter) in swift current, often in rivers with bedrock substrate.

### Known distribution.

South Africa.

## Oligoneuriopsis dobbsi

Taxon classificationAnimaliaEphemeropteraOligoneuriidae

(Eaton, 1912)

2943C59E-1D65-5DAA-913C-218785B2B626

Figures 5 , 6

Oligoneuria dobbsi Eaton, 1912: 243, fig. 1 (female imago).
Oligoneuriella dobbsi : Ulmer, 1924: 32.
Oligoneuria sp.: Vayssière, 1936: 130 (nymph).
Oligoneuriopsis dobbsi : Kimmins, 1960: 276, figs 9, 10 (female and male imagos).
Oligoneuriopsis grandaeva (Navás, 1936: 125, fig. 21) (female imago).

### Material examined.

Kenya • 28N; Mount Elgon, Teremi upstream; 0.8973°N, 34.5973°E; alt. 2456 m a.s.l.; 13 Oct. 2019; W. Graf leg. • 23N; Mount Elgon, Teremi; 0.9094°N, 34.5994°E; alt. 2407 m a.s.l.; 13 Oct. 2019; W. Graf leg. • 13N; Mount Elgon, Kimurio upstream; 0.8913°N, 34.5892°E, alt. 2239 m a.s.l.; 11 Oct. 2019; W. Graf leg. • 46N (among them 1N – GBIFCH00890747 – sequenced); Mount Elgon, Kimurio tributary 2; 0.8956°N, 34.5878°E; alt. 2347 m a.s.l.; 8 Nov. 2019; W. Graf leg. • 15N; Mount Elgon, Kibisi upstream; 0.9028°N, 34.6175°E; alt. 2298 m a.s.l.; 9 Nov. 2019; W. Graf leg. • 8N; Mount Elgon, Kapkateny upstream; 0.8959°N, 34.5990°E; alt. 2293 m a.s.l.; 11 Oct. 2019; W. Graf leg. • 7N; Mount Elgon, Kapkateny midstream; 0.8325°N, 34.6234°E; alt. 1896 m a.s.l.; 12 Oct. 2019; W. Graf leg. • 2N; Mount Elgon, Kapkateny downstream;0.8144°N, 34.6243°E; alt. 1660 m a.s.l.; 14 Oct. 2019; W. Graf leg.; all MZL.

### Male imago.

**Figure 5.**
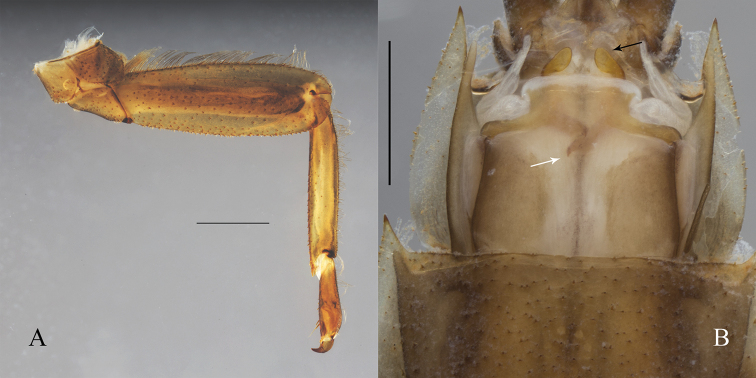
*Oligoneuriopsis dobbsi*, male nymph **A** hind leg **B** genitalia (sternite IX removed). Black arrows: Apex of the lateral longitudinal lobe of penis. White arrows: Proximal process of penis. Scale bar: 1 mm.

As redescribed by Kimmins (1960), with the following complement extracted from mature male nymphs: penis lobes with characteristic sclerotized proximal process ending in a simple projection; apex of the lateral longitudinal lobe of penis in a small club-shaped sclerite ca. 1.5 × larger than the lateral lobe (Fig. 5B).

### Nymph.

Lengths. Body up to 17 mm and 25 mm in male and female nymphs respectively; cerci (and caudal filament) up to 10 mm and 11 mm in male and female nymphs, respectively.

**Figure 6.**
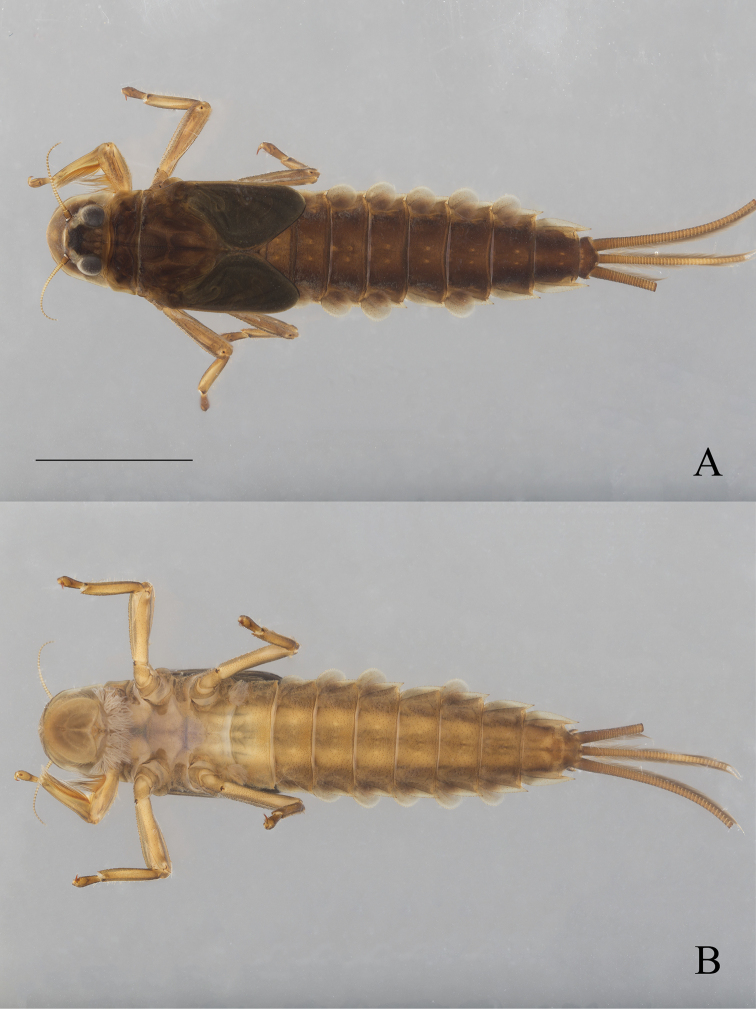
*Oligoneuriopsis dobbsi*, male nymph **A** in dorsal view **B** in ventral view. Scale bar: 5 mm.

General colouration medium to dark brown, in general darker in mature nymphs than in immature ones (Fig. 6A). Head dark brown, with four lighter maculations between the compound eyes, and a black marking present on the frons between the ocelli; generally also with a rounded light maculation between antennae. Ventrally, head a uniform light brown colour. Gills at base of maxillae forming a “beard” ventrally at base of head, much paler in colour relative to head capsule (Fig. 6B). Pro- and mesonotum dark brown, with lighter maculae laterally and medially. Legs light to medium brown, femoro-tibial articulation with a blackish spot. Femur and tibia of foreleg shorter than those of mid or hind leg, fore tibia longer than fore femur; on mid- and hind legs, femora and tibiae subequal in length. Setae on the outer margin of mid and hind femora well developed, slightly decreasing in size and reaching the apex (Fig. 5A). Tibiae and tarsi with long, even fringe of setae along entire dorsal margin. Abdominal tergites uniformly dark brown, each with a pair of light spots in the middle, except tergite X which bears four light spots in proximal part. Sternites medium brown, laterally dark brown, with two small pale median markings, especially visible on sternites IV to VIII. Dense patch of posteriorly orientated setae ventromedially moderately developed on abdominal sternite II, well-developed on sternites III–V, absent from other segments. Gills III–VII almost subequal in size, more than ¾ of the corresponding segment, gill II smaller, ca. 1/2 the size. On all gills except for gill I, fibrillae shorter than lamella length. Lamella of gill I a little bit shorter than the length of the fibrillar portion. Lamellae II–VII with long and thin setae on their distal inner margin. Posterolateral spines of the abdomen absent of segments I and II, then increasing in size from segment III to IX, those of this last one being ca. ¼ the length of the segment.

Cerci uniformly medium brown, caudal filament paler brown towards apex.

### Affinities.

*Oligoneuriopsis dobbsi* male imago seems to be closely related to *O. lawrencei* from which it differs by the presence of crossveins in the proximal part of the subcostal area (see Kimmins 1960, fig. 9), as well as by the shape of the proximal process of the penis sclerite which is shorter and less pointed than in *O. lawrencei*. The supposed nymph of *O. dobbsi* presents also similarities with the one of *O. lawrencei*, but differs in several respects, namely, the absence of a slight carina on the head, the general colouration of the body, the size of gill II, smaller than the following ones in *O. dobbsi*, whereas subequal to the following ones in *O. lawrencei*, the patch of setae on sternites (II) III–V in *O. dobbsi* compared to sternites II–IV (V–VI) in *O. lawrencei*, and finally the size of gill I lamella, much shorter in the latter than in *O. dobbsi*.

### Remarks.

The association of the nymphs from Mount Elgon with the adults described by Eaton (1912) as *Oligoneuriopsis dobbsi* is putative at the moment, because we have no rearing of the nymphs and no COI sequences from Eaton’s material. However, we think this association is realistic, for the following reasons. First, male genitalia extracted from a mature nymph are compatible with those drawn by Kimmins (1960), especially the proximal process which is thick and shorter than in the other species, and the apex of the lateral longitudinal lobe of penis which is slightly clavate distally. Secondly, localities for the nymphs and adults are only distant from ca. 100 km, whereas no other *Oligoneuriopsis* populations are known in a radius of thousands of kilometres. Additional nymphal material has been collected by Laban Njoroge, National Museums of Kenya, Nairobi, from the Aberdare range of mountains in Central Kenya and one specimen from Mount Kenya. Images of these nymphs correspond completely with the nymphs of *O. dobbsi* described here. The Aberdare range is ca. 200 km east of the type locality, Kericho, while Mount Elgon is around 150 km north. It is reasonable to assume that a single species is represented in this area.

### Known distribution.

Kenya.

## Oligoneuriopsis jessicae

Taxon classificationAnimaliaEphemeropteraOligoneuriidae

Agnew, 1973

CDC33762-33AA-5704-8077-53FD37EC01F6

Figure 7

Oligoneuriopsis jessicae Agnew, 1973: 116, fig. 1A, B (nymph).

### Material examined.

Eswatini (former Swaziland) • 14N; Malolotja stream, Nkomati River system; 26.1167°S, 31.1144°E; alt. 1227 m a.s.l.; 3 Mar. 2003; R. Bills leg.; AMGS; GEN 1733E • 7N; Jubukweni stream near Mbuluzi, Nkomati River system; 26.2028°S, 31.1944°E; alt. 1044 m a.s.l.; 29 Mar. 2003; R. Bills leg.; AMGS; GEN 1734B • 4N; Lubuyane stream near Mnyokane, Nkomati River system; 26.1572°S, 31.2081°E; alt. 1435 m a.s.l.; 4 Apr. 2003; R. Bills leg.; AMGS; GEN 1738B.

### Comments.

Agnew (1973) examined material from the National Institute for Water Research (NIWR), Pretoria, and stated it would be housed in the Transvaal Museum (now known as Ditsong Museum). However, Agnew (pers. comm., 1983) indicated that when he moved from the university where he had been based, the technicians in his former laboratory discarded all of the material that he had left in his office, including all of the Oligoneuriidae material he had examined. Examination of mayfly material in Ditsong Museum (February 2019), and discussions with the late Curator Dr Martin Kruger^†^ (pers. comm., 22 February 2019) confirmed that this material was not in this museum. As no material has since been collected from or near to the type locality (Queen River, 35 km from Barberton, 25.8200°S, 30.8100°E), no neotype has been designated.

### Male and female imagos.

Unknown.

### Nymph.

**Figure 7.**
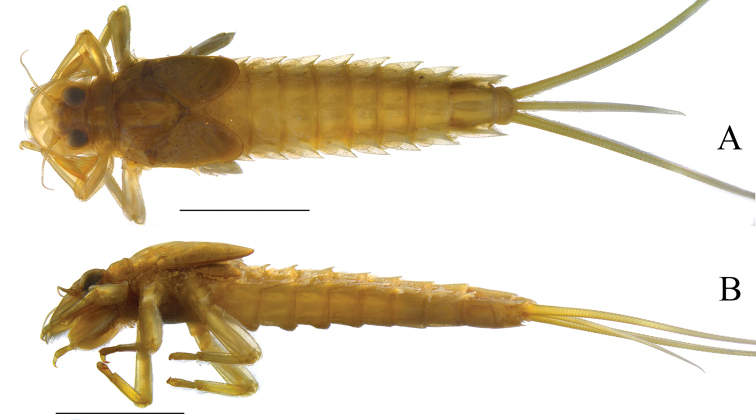
*Oligoneuriopsis jessicae***A** whole nymph, dorsal view **B** lateral profile, showing dorsal abdominal tubercles. Scale bar: 5 mm (**A**).

Lengths. Body up to 20 mm and 22 mm in male and female nymphs respectively; cerci (and caudal filament) up to 14 mm (4.5 mm) and 15.8 mm (6.9 mm) in male and female nymphs respectively. General colouration pale to hazelnut brown, with small, paired paler spots in the middle of each tergum of mature nymphs in some individuals; head pale to dark brown, without markings, darker between eyes, becoming paler brown towards distal margin of head (Fig. 7A). Ventrally, head chestnut brown, gills at base of maxillae forming a pale cream coloured “beard” ventrally at base of head. Pro- and mesonotum dark brown, with pale brown marking on mesonotum, forming a distinct M-shape in mature nymphs. Legs light brown, femoro-tibial articulation darker; setae of forelegs light brown, same colour as the legs. Femur and tibia of foreleg shorter than those of mid or hind leg; in all cases, femora longer than tibiae. Coxal-femoral articulation of mid and hind legs with dark brown stripe ventrally. Setae on the outer margin of mid and hind femora well developed, tapering off slightly in length towards the apex, scattered spatulate setae over entire surface but more concentrated along margins; mid and hind tibae and tarsi with strong fringe of fine, even setae along the outer margin. Distal end of tibiae of mid legs with three stout spines on inner side.

Abdominal tergites darker than sternites, no distinctive markings except for dorsal paired paler brown spots on each side of the midline of the tergites in mature specimens; sharply pointed dorsal tubercles present on tergites I–VII, gradually decreasing in size posteriorly (Fig. 7A); sternites uniform pale brown, with no markings. Dense patch of posteriorly orientated setae ventromedially on abdominal segments II–V, much reduced patch on segment VI. Gills II–VII almost subequal in size, gill I smaller. On all gills except for gill I, fibrillae slightly shorter than lamella length. Lamella of gill I less than half the length of the fibrillar portion. Lamellae II–VII with long and thin setae on their distal inner margin. Posterolateral spines of the abdomen increasing in size posteriorly. Cerci and caudal filament uniformly medium brown.

Whole nymph (dorsal aspect) and gills as illustrated by Agnew (1980) and Fig. 7A. Lateral view of anterior of nymph (Fig. 7B), shows dorsal abdominal spines in profile.

### Affinities.

Nymphs of *O. jessicae* mainly differ from those of *O. lawrencei* by the presence of sharply pointed dorsal tubercles on tergites I–VII. The dorsal setae along the hind femur are long in *O. lawrencei*, *O. dobbsi* and *O. elisabethae*, extending to the apex of the femur, while in *O. jessicae* the setae are shorter and taper off, not reaching the apex. Gills in *O. jessicae* are shorter than the half length of the corresponding tergite, as in *O. elisabethae*, while in *O. lawrencei*, the gills reach approximately half length of the corresponding tergite, even longer in *O. dobbsi*. Adult material is needed for comparison with other species.

### Habitat preference.

Moss-covered stones in current. Nymphs mature in April (autumn).

### Known distribution.

Eswatini; South Africa: Mpumalanga near Barberton.

## Oligoneuriopsis elisabethae

Taxon classificationAnimaliaEphemeropteraOligoneuriidae

Agnew, 1973

FF66993A-CFCC-5DF0-B63F-A255E3C79DA8

Figure 8

Oligoneuriopsis elisabethae Agnew, 1973: 118, fig. 1C (nymph).

### Material examined.

Lesotho • 4N; Tsoelikana River; 29.9200°S, 29.0925°E; alt. 2247 m a.s.l.; 21 Jan. 1986; K. Meyer; AMGS; LES 38U • South Africa • 4N; KwaZulu-Natal, Umkomozana River, Sani Pass; 29.5842°S, 29.2883°E; alt. 2870 m a.s.l.; 14 Jan. 2011; T.A. Bellingan; AMGS; GEN 1978C.

### Comments.

As with *O. jessicae*, the material examined by Agnew has been lost. This includes VAL 606G Klip River, Vrede-Volksrust Rd. Bridge 1959/01/14 27.35806°S, 29.35000°E, Free State Province, and VAL 1061B, VAL 1062A. Klein Vaal R., at Goedehoop Farm 1960/03/22 26.8194°S, 30.1333°E, Mpumalanga Province, F.M. Chutter leg. As the material examined is from a different catchment to the type material, no neotype has been designated.

### Male and female imagos.

Unknown.

### Nymph.

**Figure 8.**
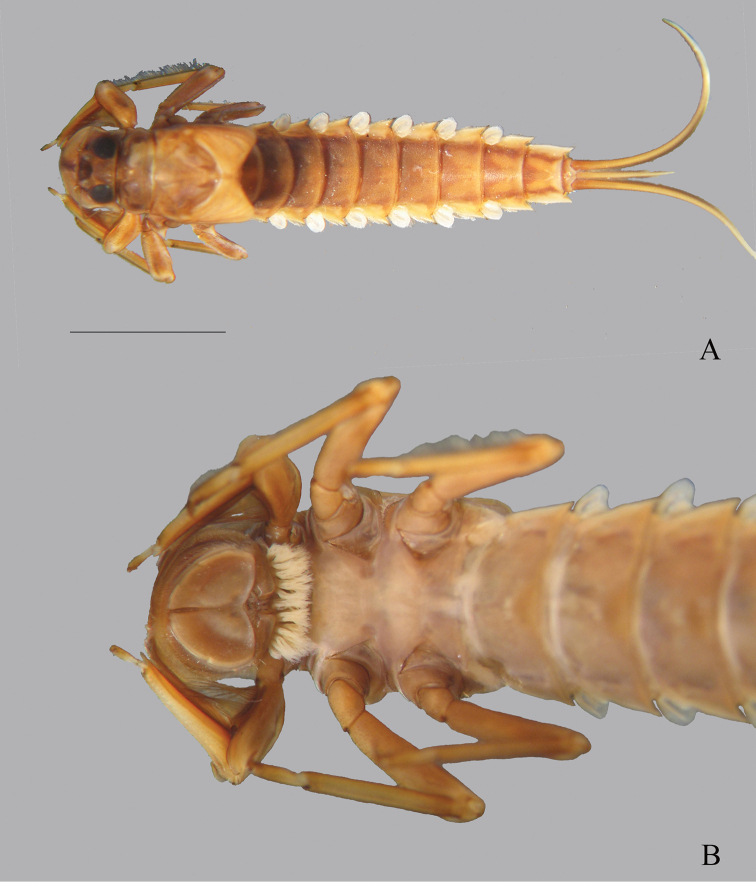
*Oligoneuriopsis elisabethae***A** whole nymph (immature), dorsal view **B** dorsal view of head. Scale bar: 5 mm (**A**).

Lengths. Body up to 16.5 mm (Agnew 1973), sex-based size differences of nymphs not recorded as none of the available material is fully mature. Cerci up to 8.1 mm, caudal filament 3.3 mm. General colouration light brown (Fig. 8A). Head (Fig. 8A) light brown, with darker brown marking between bases of antennae and ocelli and a grey-brown maculation between antennae; carina on frons slightly developed. Ventrally, head a uniform light brown colour; gills at base of maxillae forming a “beard” ventrally at base of head, much paler in colour relative to head capsule (Fig. 8B). Pro and mesonotum pale brown, with darker maculae laterally. Legs light brown, femoro-tibial articulation with a blackish spot. Femur and tibia of foreleg shorter than those of mid or hind leg; femora and tibiae of mid and hid legs of approximately equal length. Setae on the outer margin of mid and hind femora well developed, evenly distributed along length of margin; mid and hind tibiae and tarsi with well-developed fringe of even setae along the outer margin. Middle and hind legs with basal area of coxae and trochanter, and entire surface of femora covered with small, scattered, dark brown spine-like setae, extending also along ventral margins of tibiae and tarsi, as well as interspersed amongst the fringe of setae along the dorsal margins.

Abdominal tergites uniformly pale brown, no distinctive patterns except for paired pale cream-coloured markings forming a V-shaped pattern on last three abdominal segments in some specimens (Fig. 8A). Sternites uniform pale brown, with no distinctive markings (Fig. 8B). Dense patch of posteriorly orientated setae ventromedially moderately developed on abdominal sternite II, well-developed on sternites III–V, absent from other segments. Gills paler in colour than abdomen, gill I ventrally orientated, lamella less than one third the length of the fibrillar portion, gills II–VII with rounded lamella, filaments shorter than the corresponding lamella, lamella shorter than half the length of the corresponding segment. Lamellae II–VII with long and thin setae on their distal inner margin. Posterolateral spines of the abdomen of similar size on each segment, each with the tip a darker brown. Cerci uniformly medium brown, caudal filament paler brown towards apex, less than half the length of cerci.

### Affinities.

Nymphs of *O. elisabethae* are less flattened compared to *O. lawrencei*, and have the shortest gills relative to the abdominal tergite length of the three South African species, at ca. 1/3 of the length of the tergites. Head similar in shape to *O. jessicae* but notably different to *O. lawrencei*, which is widest medially. Lateral abdominal spines are well developed; dorsal abdominal spines as seen in *O. jessicae* are absent in *O. elisabethae.* Nymphs of *O. elisabethae* also differ from those of *O. skhounate*, *O. dobbsi*, *O. orontensis* and *O. villosus* by the reduction of the caudal filament.

### Habitat preference.

Found in cobble, pebble and gravel substrate in swift current.

### Known distribution.

Lesotho; South Africa: Free State and Mpumalanga Provinces.

## Oligoneuriopsis skhounate

Taxon classificationAnimaliaEphemeropteraOligoneuriidae

Dakki & Giudicelli, 1980

295BDABD-9C9D-5054-BD1F-79A1E4D2DFB4

**Figure 9.**
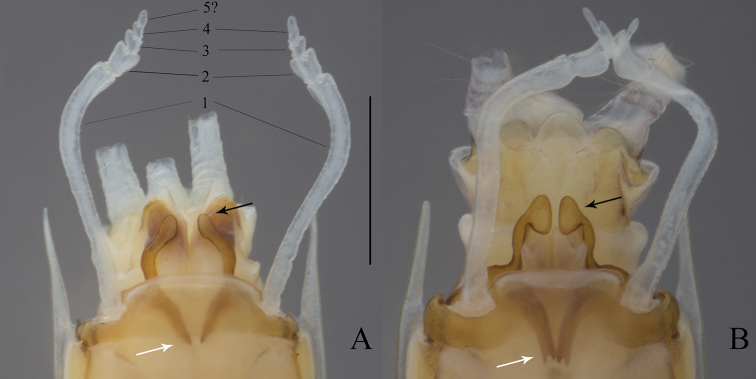
Genitalia of *Oligoneuriopsis* in ventral view **A***O. skhounate***B***O. orontensis*. Black arrows: Apex of the lateral longitudinal lobe of penis. White arrows: Proximal process of penis. Scale bar: 1 mm.

**Figure 10.**
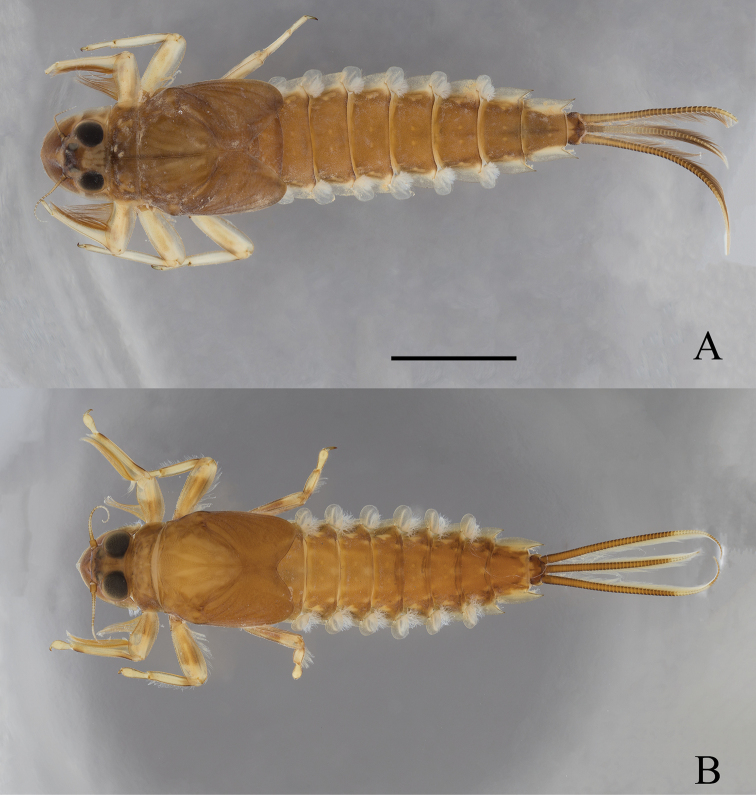
Habitus of *Oligoneuriopsis* nymphs in dorsal view **A***O. skhounate***B***O. orontensis*. Scale bar: 5 mm.

**Figure 11.**
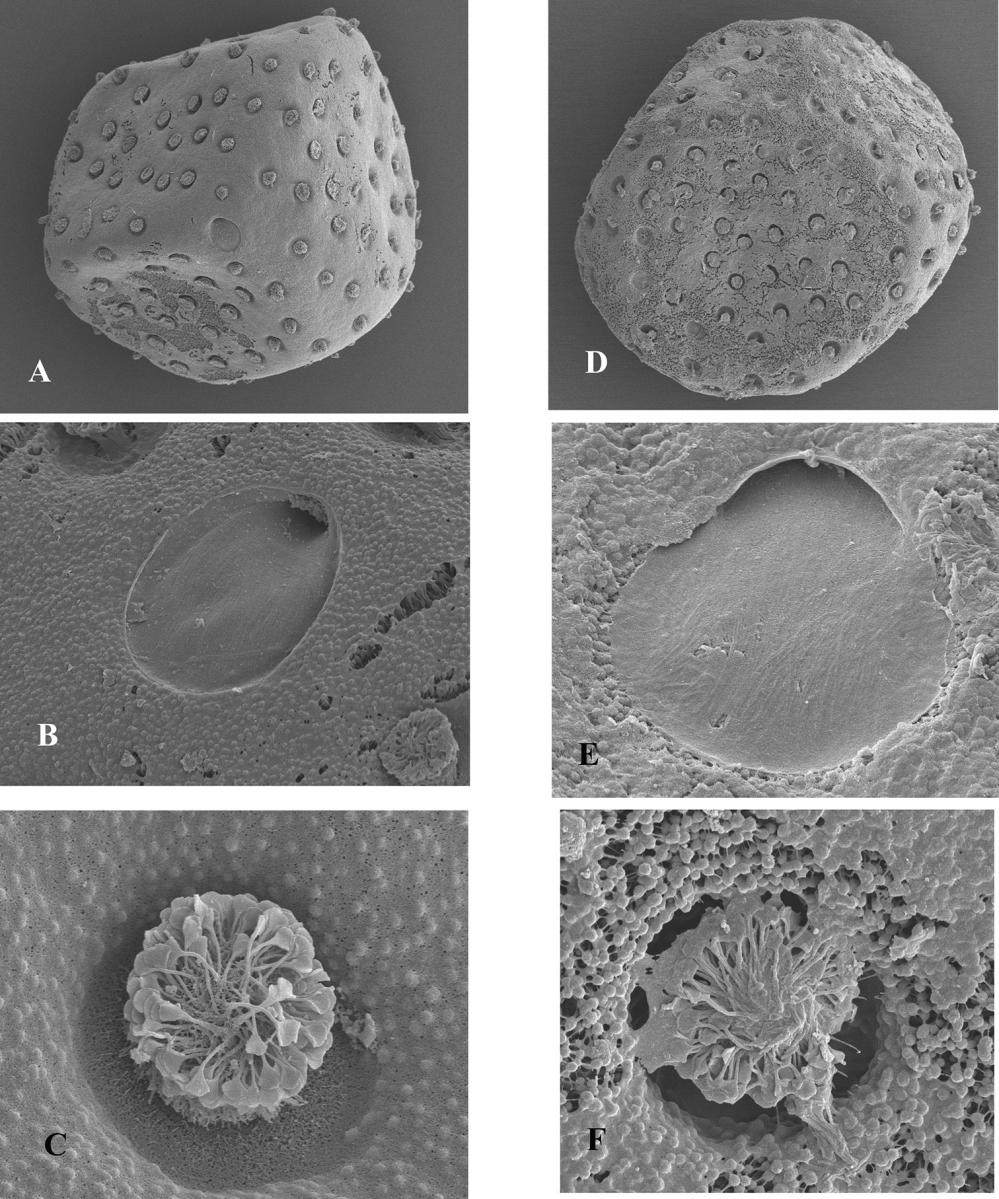
*O. skhounate* (**A–C**) and *O. orontensis* (**D–F**) **A, D** shape of the egg **B, E** micropyle **C, F** KCT’s.

**Figure 12.**
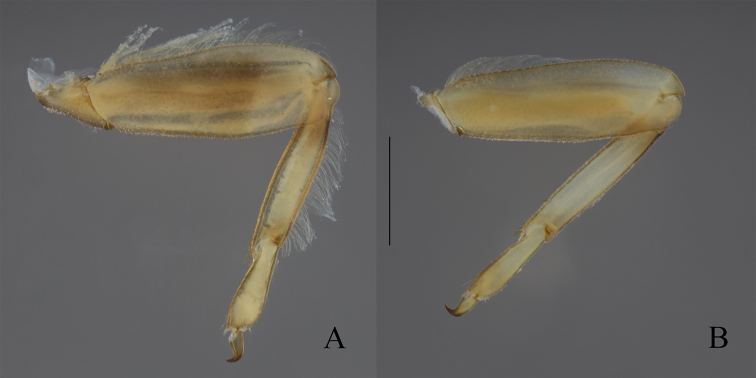
Hind legs of *Oligoneuriopsis* in dorsal view, showing femoral and tibial setation **A***O. skhounate***B***O. orontensis*. Scale bar: 1 mm.

Figures 9A , 10A , 11A–C , 12A , 14E , 15E

Oligoneuriopsis skhounate Dakki & Giudicelli, 1980: 19, figs 14–29 (male imago, nymph).

### Material examined.

Algeria • 1N (sequenced GBIFCH00763571); Oued Cherf, Medjez Amar; 36.44306°N, 7.31083°E; alt. 205 m a.s.l.; 3 Oct. 2018; B. Samraoui leg.; MZL • 2N (sequenced GBIFCH00763569–GBIFCH00763570); Oued Cherf, Dbabcha; 36.2166°N, 7.3181°E; alt. 610 m a.s.l.; 18 Oct. 2019; B. Samraoui leg.; MZL • Morocco • 3N; Marrakech, Palmeraie, Oued Tensift; 31.6619°N, 7.9694°W (estimated); alt. 443 m a.s.l.; 27 Apr. 1960; J. Aubert leg.; MZL • Spain • 4N; Pyrénées, Barbastro (Huesca), Rio Vero; 42.2400°N, 0.1278°W (estimated); alt. 1000 m a.s.l.; 24 Jun. 1956; H. Bertrand leg.; MZL • 1N; Sierra Morena, Venta de Cardenas; 38.4006°N, 3.5119°W (estimated); alt. 650 m a.s.l.; 2 Aug. 1960; J. Aubert leg.; MZL • 3N; Valladolid, Cabezon, Rio Pisuerga; 41.4650°N, 5.2297°W (estimated); alt. 650 m a.s.l.; 17 Aug. 1988; D. Studemann & P. Landolt leg.; MZL • 41N; Malaga, Cortes de la Frontera, Rio Guadairo; 36.5483°N, 5.3675°W (estimated); alt. 250 m a.s.l.; 21 Aug. 1988; D. Studemann & P. Landolt leg.; MZL • 4N, 10s♀, 17♂; same locality; 15 Sep. 1988; P. Landolt leg.; MZL • Tunisia • 1N; Bizerte, Mateur, Oued Joumine, upstream Lake Ichkeul dam; 36.9628°N, 9.5244°E; alt. 105 m a.s.l.; 20 Nov. 2004; S. Zrelli leg.; LBE • 22N, 1s♀; same locality; 26 Jun. 2005; S. Zrelli leg.; LBE • 20N, 2s♀; same locality; 18 Jul. 2005; S. Zrelli leg.; LBE • 40N; same locality; 28 Aug. 2005; S. Zrelli leg.; LBE • 40N; same locality; 6 Sep. 2005; S. Zrelli leg.; 39 LBE, 1 MZL • 40N; same locality; 24 Oct. 2005; S. Zrelli leg.; LBE • 35N; same locality; 26 Jun. 2006; S. Zrelli leg.; 24 LBE, 11 MZL • 11N; same locality; 31 Jul. 2006; S. Zrelli leg.; LBE • 2N, 1s♀; same locality; 6 Apr. 2009; S. Zrelli leg.; MZL • 2N; same locality; 17 May 2010; S. Zrelli leg.; LBE • 5N; Tabarka, Oued Bouterfes; 36.953°N, 8.9125°E; alt. 100 m a.s.l.; 4 Jan. 2005; S. Zrelli leg.; LBE • 6N; Jandouba, Fernana, Oued Ellil; 36.7203°N, 8.7339°E; alt. 237 m a.s.l.; 28 Jul. 2005; S. Zrelli leg.; LBE • 2N; same locality; 12 Sep. 2005; S. Zrelli leg.; LBE • 4N; same locality; 29 Jul. 2006; S. Zrelli leg.; LBE • 9N; same locality; 30 Aug. 2006; S. Zrelli leg.; LBE • 10N; same locality; 26 Jun. 2008; S. Zrelli leg.; LBE • 7N; Oued Ghezala; 36.6431°N, 8.6986°E; alt. 229 m a.s.l.; 30 Aug. 2006; S. Zrelli; LBE • 5N; same locality; 21 Nov. 2009; S. Zrelli; LBE.

### Male imago.

Adequately described and illustrated by Dakki and Giudicelli (1980). The most important character is on the genitalia where the shape of the lateral longitudinal lobe of the penis ends in a rounded sclerite a little bit larger than the lateral lobe (Fig. 9A).

### Nymph

**(Fig. 10A).** Adequately described and illustrated by Dakki and Giudicelli (1980), with the following complements: setae on the outer margin of hind femora well developed and reaching the apex; outer margin of hind tibiae covered by a dense row of long and thin setae; lamella of gill I minute, fibrillae much longer than the lamella length; setae on distal inner margin of gills II–VII short and thin; posteromedially sternal patch of long setae present on segments (II)III–V, most developed on segments III–IV.

### Eggs.

General shape rhomboid, ca. 280 µm long and 250 µm wide, (Fig. 11A) chorionic surface rather smooth, micropyle tagenoform, smooth, sperm guide well apparent (Fig. 11B), KCT’s rather regularly arranged, ca. 10 µm of diameter, formed by coil-thread ending in a leaf-like and flat structure (Fig. 11C).

### Affinities.

At the male adult stage, *O. skhounate* is distinguished from *O. lawrencei* by the presence of crossveins in the proximal part of the subcostal area, and from *O. lawrencei* and *O. dobbsi* by the shape of the apex of the lateral longitudinal lobe of penis sclerite which is only slightly enlarged. In the nymphal stage, *O. skhounate* differs from *O. jessicae* by the absence of abdominal carina and from *O. lawrencei* and *O. elisabethae* by the setation of the dorsal margin of hind femora with much longer setae; it also differs from *O. dobbsi* by the size of gill I lamella, much longer in the latter.

The ecology of the nymph in North Africa is described by Bouhala et al. (2020).

### Known distribution.

Algeria, Morocco, Spain, Tunisia.

## Oligoneuriopsis orontensis

Taxon classificationAnimaliaEphemeropteraOligoneuriidae

(Koch, 1980) comb. nov.

71EEB0A8-E519-57C4-9176-844730E66534

Figures 9B , 10B , 11D–F , 12B , 14F

Oligoneuriella orontensis Koch, 1980: 154, figs 1–4 (nymph).

### Material examined.

Israel • 1N; Jordan River, Ateret Fortress; 33.0032°N, 35.6286°E; alt. 63 m a.s.l.; 7 May 1990; H. Glassmann & M. Sartori leg.; MZL • 1N; same locality; 7 May 1991; H. Glassmann & M. Sartori leg.; MZL • 5N; same locality; 8 Dec. 2014; Z. Yanai leg.; SMNH • 15N, 3♂; same locality; 29 Oct. 2015; Z. Yanai & Y. Brenner leg.; SMNH • 14N; same locality; 16 May 2016; Z. Yanai & A. Charvet leg.; SMNH • 8N; same locality; 2 Jun. 2016; Y. Hershkovitz & T. Eshcoly leg.; SMNH • 3N (1N – GBIFCH00759464 – sequenced); same locality; 11 Mar. 2017; Z. Yanai & J.-L. Gattolliat leg.; MZL, SMNH • 4N (2N – GBIFCH00664952, GBIFCH006759463 – sequenced); same locality; 27 Mar. 2019; Z. Yanai leg.; MZL, SMNH • 2N; Dan Stream, st. 6; 33.1320°N, 35.3845°E; alt. 120 m a.s.l.; 10 May 1990; A. Reuven & M. Sartori leg.; MZL • 4N; Senir (Hasbani) Stream, upstream Ma’ayan Barukh Bridge; 33.2253°N, 35.6152°E; alt. 103 m a.s.l.; 10 May 1990; A. Reuven & M. Sartori leg.; MZL • 1N; same locality; 10 May 1991; A. Reuven & M. Sartori leg.; MZL • 2N; Senir (Hasbani) Stream, downstream ‘En Barukh; 33.2308°N, 35.6209°E; alt. 119 m a.s.l.; 10 May 1990; A. Reuven & M. Sartori leg.; MZL • 2s♀, 1♂; same locality; 25 Jul. 1990; A. Reuven leg.; MZL • 4s♀, 3♂; same locality; 10 May 1991; A. Reuven & M. Sartori leg.; MZL • 4N; Hermon (Banyas) Stream, Kefar Szold; 33.1301°N, 35.3832°E; alt. 100 m a.s.l.; 8 May 1991; A. Reuven & M. Sartori leg.; MZL • 1N; Jordan River, haDodot Bridge; 32.9318°N, 35.6226°E; alt. -161 m a.s.l.; 29 Jul. 2015; Y. Hershkovitz & T. Eshcoly leg.; SMNH • 2N; Jordan River, Park haYarden; 32.9091°N, 35.6235°E; alt. -203 m a.s.l.; 2 Jun. 2016; Y. Hershkovitz & T. Eshcoly leg.; SMNH • 2s♂; Senir (Hasbani) Stream, Beth Hillel; 33.1989°N, 35.6108°E; alt. 82 m a.s.l.; 6 Aug. 2020; Z. Yanai & A. Hershko leg.; SMNH.

### Male imago.

Lengths. Body: up to 18 mm; forewing: up to 17 mm; cerci: up to 15 mm; caudal filament: up to 13 mm.

Vertex light brown, frontoclypeus yellowish, compound eyes greyish black, base of ocelli black, ocelli whitish, antennae with pedicel light brown and flagellum medium brown.

Pronotum light brown, washed with grey. Pterothorax light brown, with a large mesonotal suture yellowish. Forelegs with outer margin of femora, tibiae and tarsi medium brown, inner margin yellowish; mid- and hindlegs yellowish, with distinct inner brown maculae on the femoro-tibial articulation; wings, when folded, light brown, almost whitish when unfolded.

Abdominal segments uniformly yellowish, without distinct patterns, except sides of tergite IX, tergite X and sternite IX light brown; gonostyli and cerci whitish.

Forelegs functional, tibiae and tarsi of middle and hind legs weakly sclerotized and non- functional. Tarsal claws blunt. Forewing typical of the genus, with 5 groups of veins: Sc+RA, RSa+iRS, RSp+MA1, MA2+MP1, and a forked MP2+CuA – CuP vein. Subcostal field with numerous transversal veins, those issued from RA not reaching iRS in the distal forth of the length, those between iRS and MA1 only present in the proximal half.

Gonostyli 4-segmented, the basal one ca. 4 × the length of segments 2 to 4 combined; a fifth segment can sometimes be present (Fig. 9B). Penis lobes with characteristic sclerotized proximal process ending in a bifid projection; lateral longitudinal lobe ending in a distinct club-shaped sclerite more than 2 × larger than the lateral lobe. Cerci with whorls of long setae at each junction.

### Female subimago.

Lengths. Body: up to 23 mm; forewing: up to 21 mm; cerci: up to 7 mm; caudal filament: up to 5.5 mm.

Colouration as in the male, except antennal pedicel entirely medium brown, forefemora with outer margin sepia, tibiae and tarsi of all legs atrophied, twisted on forelegs; tarsal claws reduced to a single pointed and unsclerotized filament. Cerci light to medium brown. Posterior margin of sternite IX deeply concave and rounded.

### Nymph

**(Fig. 10B).** First described by Koch (1980) sub nomen *Oligoneuriella orontensis*.

Lengths. Body up to 14 mm and 19 mm in male and female nymphs respectively; cerci (and caudal filament) up to 7 mm (5 mm) and 9 mm (7 mm) in male and female nymphs respectively.

General colouration light to medium brown, always lighter in male nymphs. Head medium brown, yellowish between the compound eyes. Pronotum medium brown, with yellowish areas sublaterally. Pterothorax medium brown, with yellowish maculae very characteristic (see Koch, 1980, fig. 4). Legs light brown, femora medium brown in the proximal half, lighter distally; femoro-tibial articulation darker, especially notable in mature nymphs. Setae on the outer margin of hind femora well developed, but not reaching the apex (if reaching it, then much smaller than the proximal ones) (Fig. 14F). Outer margin of hind tibiae without a row of long and thin setae (Fig. 12B). Abdominal tergites uniformly medium brown, each with a pair of light spots in the middle and one or two light maculae laterally; sternites with four spots on a transverse line and two elongated maculae anteriorly, altogether six spots creating sort of a circle (most notable in mature nymphs). Gills II–VII almost subequal in size, gill I smaller, ventral. On all gills, fibrillae shorter or subequal to lamella length. Lamellae II–VII with long and thin setae on their distal inner margin. Posteromedially sternal patch of long and thin setae present on segments II–IV(V). Posterolateral spines of the abdomen increasing in size posteriorly. Cerci uniformly dark brown, sometimes medium brown with a wide median dark band or with apex light brown.

### Eggs.

General shape rhomboid, ca. 300 µm long and 270 µm wide, chorionic surface finely granulated (Fig. 11D), micropyle tagenoform, smooth, sperm guide well apparent (Fig. 11E), KCT’s rather regularly arranged, ca. 10 µm of diameter, formed by coil-thread ending in a leaf-like and flat structure (Fig. 11F).

### Affinities.

In male imagos, *O. orontensis* differ from all other known species by the apex of the lateral sclerite of the penis, which is greatly enlarged, even more than in *O. dobbsi*, and the proximal process of the penis which is bifid. Nymphs are characterized by a row of setae on the outer margin of hind femora which does not reach the apex compared to other species studied, except *O. jessicae* to some extent, and differs to all other known species by the absence of a row of setae on hind tibiae.

**Figure 13.**
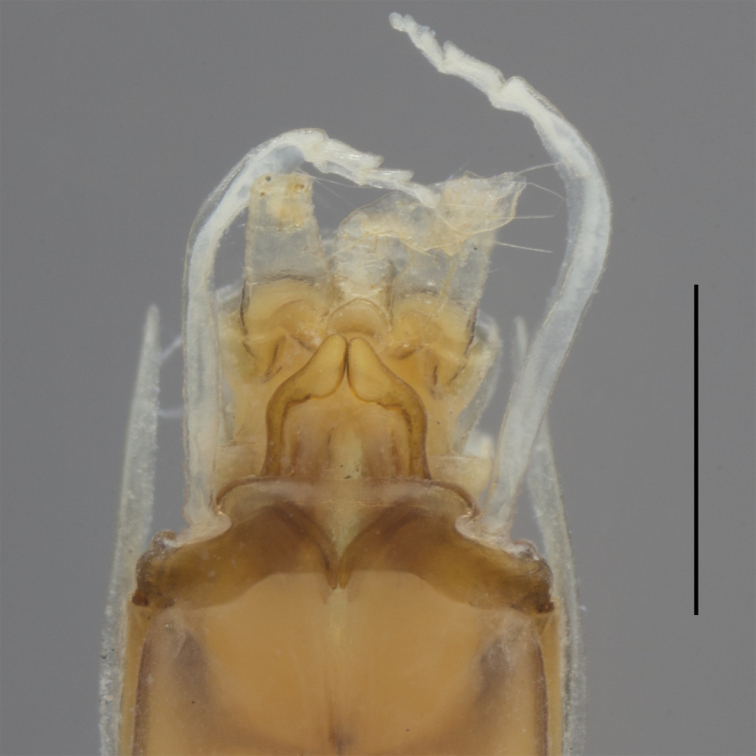
Genitalia of *Oligoneuriopsis* sp. from Iran in ventral view. Scale bar: 1 mm.

### Habitat preference.

In Israel, found in well-oxygenized streams with high water discharge and current velocity (Yanai et al. 2020). The scarcity of these habitats in the Levant may be the reason for its recent decline in Israel, and perhaps in other countries, although no recent data are available.

### Known distribution.

Israel, Lebanon, Syria, Turkey.

### Comments.

Al-Zubaidi and Al-Kayatt (1986) reported on “*Oligoneuriopsis* sp.” from northern Iraq (later cited by Abdul-Rassoul 2020). These individuals may belong either to *O. orontensis* or *O. villosus*, thus pushing distribution slightly eastwards or westwards, respectively. While both alternatives are possible in terms of ecology and geography, it is very likely that these specimens were misidentified and in fact belong to the genus *Oligoneuriella*, the only oligoneuriid reported by the authors in the following year (Al-Zubaidi et al. 1987). Until further information is available, we ignore this report from Iraq.

**Figure 14.**
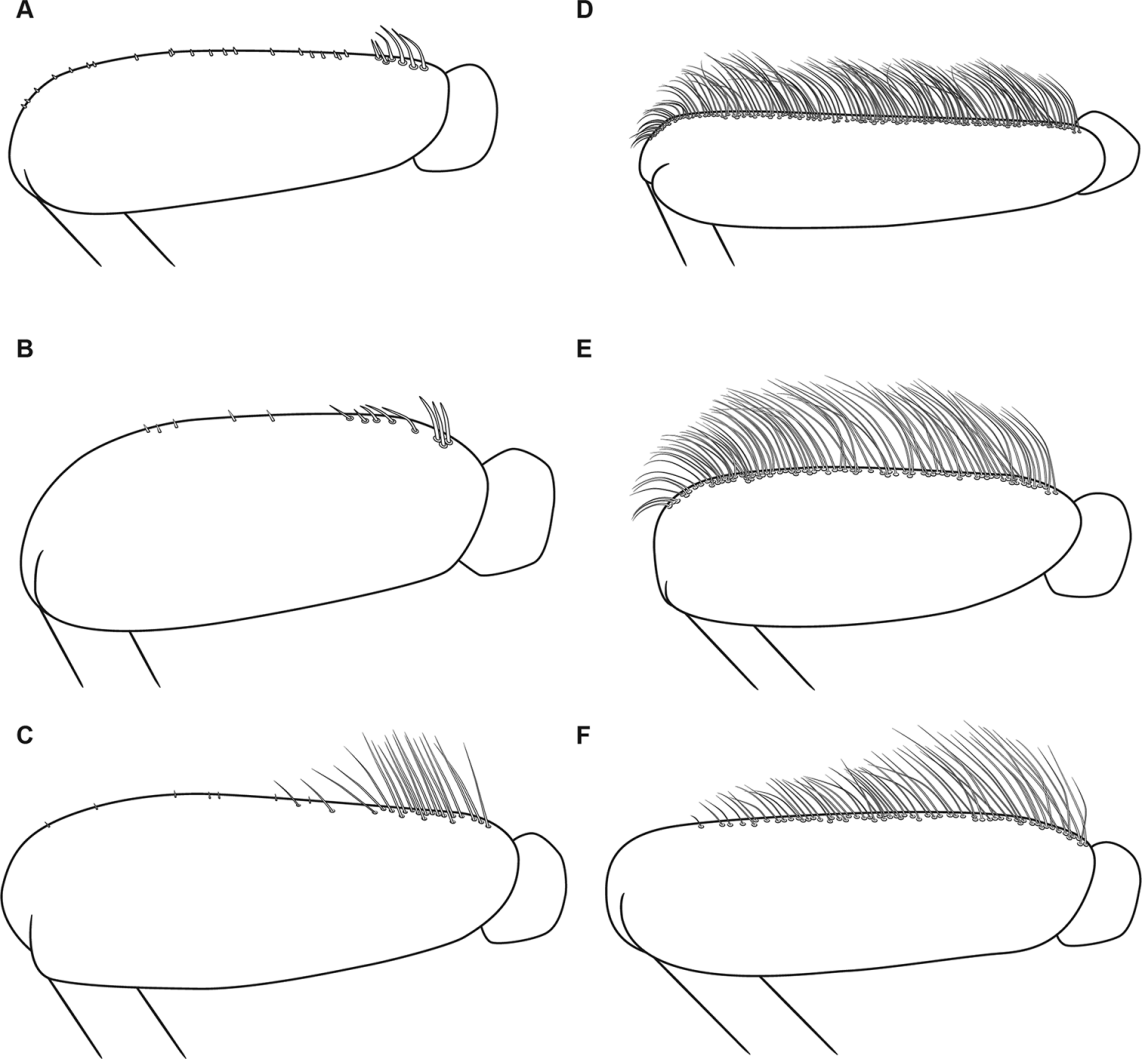
Hind femora of representative *Oligoneuriella* (**A–C**) and *Oligoneuriopsis* (**D–F**) species **A***Oligoneuriella rhenana***B***Oligoneuriella pallida***C***Oligoneuriella skoura***D***Oligoneuriopsis lawrencei***E***Oligoneuriopsis skhounate***F***Oligoneuriopsis orontensis*.

## Oligoneuriopsis villosus

Taxon classificationAnimaliaEphemeropteraOligoneuriidae

Bojková, Godunko & Staniczek, 2019

3CC55A58-256B-5B3D-86B5-F0672FD97829

Figure 15F

Oligoneuriopsis villosus Bojková, Godunko & Staniczek, 2019 in Sroka et al. 2019: 113, figs 5–8 (nymph).

### Material examined.

None.

### Male and female imagos.

Unknown.

### Nymph and eggs.

As described by Sroka et. al. (2019)

### Affinities.

The nymph of *O. villosus* can be easily differentiated from all other known species by the row of long and thin setae on the outer margin of hind femora reaching the apex, a row of long and thin setae on the outer margin of hind tibiae, posterolateral projections of abdominal segments diverging from body axis, the absence of posteromedian projections on abdominal terga, and posteromedian setae on sternites III–IV very long and dense.

### Known distribution.

Iran.

### Comment.

See comment under *O. orontensis* regarding reports from Iraq.

## Oligoneuriopsis

Taxon classificationAnimaliaEphemeropteraOligoneuriidae

sp.

EA1F4ECE-86DB-579D-9A4D-87D407D229AE

Figure 13

### Material examined.

Iran • 2♂; Ghilan, Sefid-rud River, close to Rudbar [Roodbar]; app. 36.817°N, 49.433°E; alt. 180 m a.s.l.; 4 Aug. 1972; W. Heinz coll. & V. Puthz leg. to MZL.

### Male imago.

Gonostyli 4-segmented, the basal one a little bit less than 4 × the length of segments 2 to 4 combined. Segment 2 not enlarged distally, more than 2 × longer than wide. Penis lobes with sclerotized proximal process ending in a single projection; lateral longitudinal lobe ending in a distinct club-shaped sclerite ca. 2 × larger than the lateral lobe (Fig. 13). Cerci with whorls of long setae at each junction.

### Affinities.

These two specimens are in bad state (legs missing, wings broken), but nevertheless we think it is important to report this finding. The shape of the genitalia is different from those of the previous species, somewhat intermediary between those of *O. skhounate* and *O. orontensis*. Second segment of gonostyli is also much slender than in the two previous species. These specimens could be the alate stages of *O. villosus*, but they are reported from a far distant place (ca 700 km); hence they could also belong to a new, undescribed species. Due to these uncertainties and the lack of proper material, we prefer mentioning it without naming it.

### Known distribution.

Iran.

## Revised diagnosis of *Oligoneuriopsis* Crass, 1947

**Adult.** Male gonostyli four (sometimes five)-segmented, apex of the lateral longitudinal lobe of penis club-shaped, proximal process pointed, simple or bifid; three caudal filaments, five convex longitudinal veins in forewing.

**Nymph.** Head usually without a carina, though a slight carina can be seen in some species (not pronounced as in *Elassoneuria*). Hind femora with a row of dense setae reaching at least the middle of the dorsal margin. Abdominal gills shorter than or equal to length of associated abdominal segment; apically rounded and bordered by thin setae. Postero-medial patch of very long and thin setae, well developed on sternites II to IV, sometimes to sternite V or VI.

**Generic affinities.** As already mentioned by former authors, *Oligoneuriopsis* closely resembles *Oligoneuriella* from which it can be separated by the following characters:

In *Oligoneuriopsis* male imagos, the shape of the proximal process of the penes sclerite is apically pointed or bifid, but saddle-shaped in *Oligoneuriella*, the apex of the lateral longitudinal lobe of the penis is club-shaped, enlarged at apex, but not enlarged in *Oligoneuriella*, and gonostyli are composed of four(five) segments against three (four, rarely five) in *Oligoneuriella* (including the proximal, long segment). Contrary to what Demoulin (1952) stated, there are no differences in the tarsal claw shape between the genera; they are always paired and blunt in the investigated species, as well as in all Oligoneuriidae (Kluge 2004).

**Figure 15.**
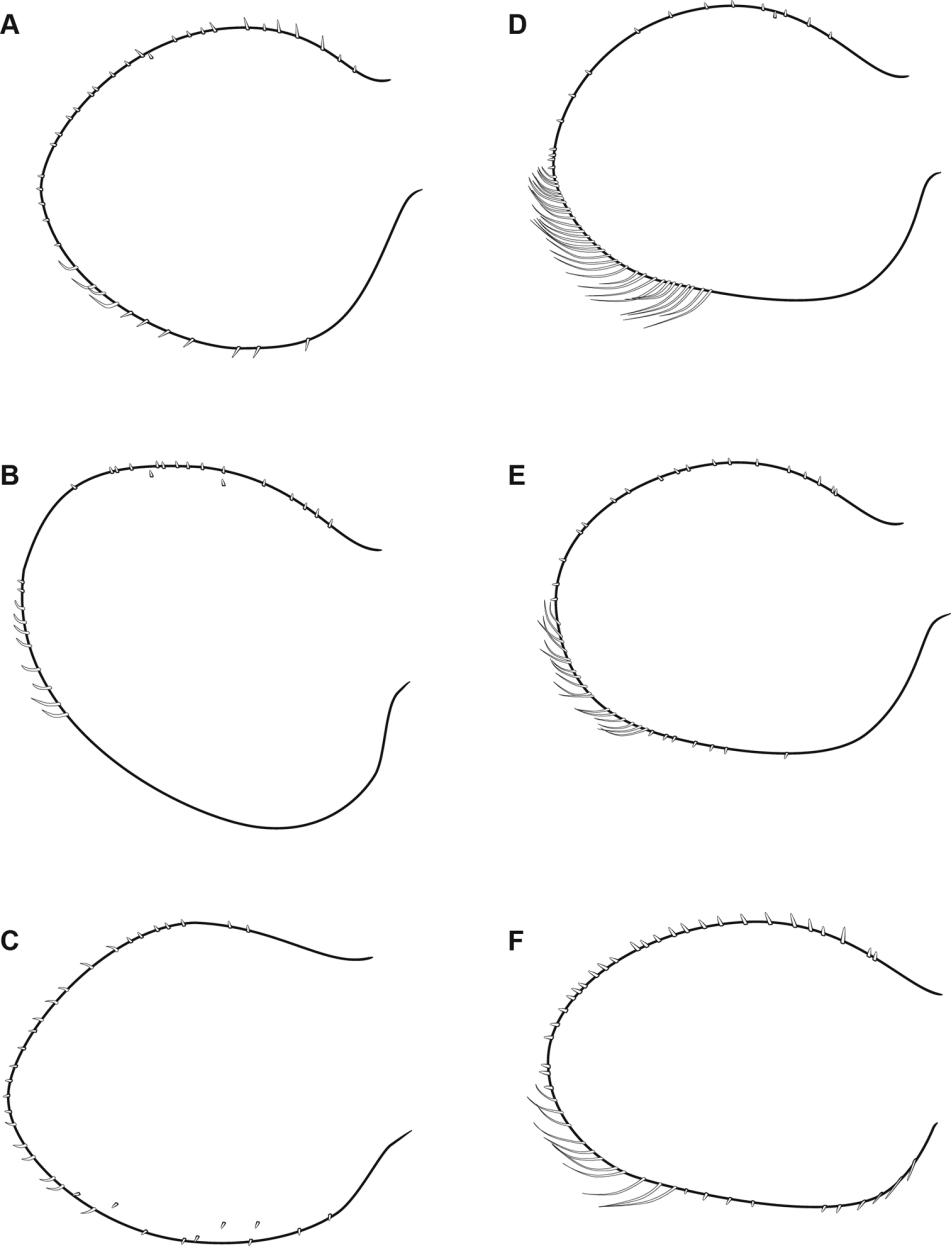
Outline of gill plate III of representative *Oligoneuriella* (**A–C**) and *Oligoneuriopsis* (**D–F**) species **A***Oligoneuriella rhenana***B***Oligoneuriella pallida***C***Oligoneuriella skoura***D***Oligoneuriopsis lawrencei***E***Oligoneuriopsis skhounate***F***Oligoneuriopsis villosus*.

In the nymphal stage, *Oligoneuriopsis* species possess a row of long and thin setae on the outer margin of the hind femora, whereas this row is absent or reduced to the proximal half of margin in *Oligoneuriella* (Fig. 14). Lamellae of gills III–V at least possess thin setae on their inner margin, whereas these setae are stout or even clavate in *Oligoneuriella* (Fig. 15). Finally, postero-medial patch of long and thin setae on proximal sternites is present in *Oligoneuriopsis*, whereas these setae and short and stout in *Oligoneuriella*.

### Key to known nymphs of *Oligoneuriopsis*

**Table:** 

1	Abdominal tergites I–VII with median tubercles (Fig. 7B)	***O. jessicae***
–	Abdominal tergites without median tubercles	**2**
2	Outer margin of hind tibiae without a row of long and thin setae (Fig. 12B)	***O. orontensis***
–	Outer margin of hind tibiae with a row of long and thin setae (e.g., Fig. 12A)	**3**
3	Caudal filament much shorter than cerci (Fig. 8A); frons slightly carinate (Fig. 8B)	***O. elisabethae***
–	Caudal filament subequal in length to cerci (e.g., Fig. 10A); frons may or may not be slightly carinate	**4**
4	Frons slightly carinate (Fig. 4B); lamella of gill I minute, abdominal marking as in Fig. 4A, although this can be less distinct	***O. lawrencei***
–	Head without carina, lamella of gill I small but well visible; abdomen without contrasted markings (e.g., Fig. 10A)	**5**
5	Posterolateral projections of the abdomen clearly divergent from body axis (Sroka et al. 2019, fig. 7A–B)	***O. villosus***
–	Posterolateral projections of the abdomen parallel to body axis (e.g., Fig. 5B)	**6**
6	Fibrillae of gill I as long as lamella; outer margin of hind tibiae with a row of rather short setae (Fig. 5A); body coloration dark brown (Fig. 6A)	***O. dobbsi***
–	Fibrillae of gill I much longer than lamella; outer margin of hind tibiae with a row of long setae (Fig. 12A); body coloration light brown (Fig. 10A)	***O. skhounate***

## Discussion

Sroka et al. (2019) proposed the presence of a row of long and thin setae on the outer margin of hind tibiae as a diagnostic character to distinguish nymphs of *Oligoneuriopsis* versus *Oligoneuriella*. Although this character is present in almost all species, it is surprisingly lacking in *O. orontensis* (Fig. 14); thus its generic value is limited.

Egg structure of *O. skhounate* and *O. orontensis* is similar to the one of *O. villosus*, as well as of *Oligoneuriella* species, but somewhat different to the one of *Oligoneuriopsis* sp. illustrated by Pescador and Peters (2007). KCT arrangement is not as dense as presented. Due to the great homogeneity in the chorionic arrangement of *Oligoneuriopsis*/*Oligoneuriella* eggs, we can suspect that the one represented by Pescador and Peters (2007) may belong to another oligoneuriid genus. In fact, it resembles much more the structure found in the Oriental oligoneuriid genus *Chromarcys* (Koss and Edmunds 1974, fig. 72).

Based on these morphological characters, a new generic concept is proposed for *Oligoneuriopsis*, meeting the need for understanding the phylogenetic position and historical development of the genus. We trust that future study of the genus (e.g., description of new species, or of unknown life stages of known species) will confirm our findings.

The COI tree (Fig. 1) obtained with novel and published Oligoneuriidae sequences clearly indicates that *O. orontensis* presents more genetic affinities with *Oligoneuriopsis* than with any other oligoneuriid taxon. This is also in accordance with the morphological analysis by Massariol et al. (2019, fig. 5) who recovered “*Oligoneuriella orontensis*” as the sister species of *Oligoneuriopsis skhounate*.

To date, seven species of *Oligoneuriopsis* are known. Four of them are described at nymphal and imaginal stages (*O. dobbsi*, *O. lawrencei*, *O. skhounate*, *O. orontensis*), and three only at the nymphal stage (*O. jessicae*, *O. elisabethae*, *O. villosus*). An additional, undescribed, species from Iran is mentioned here (Table 2). It is very likely that more African species will be found in future, for example, in the highlands of Zimbabwe, Mozambique, Malawi, and Tanzania, where very little research has yet been carried out.

**Table 2.** Available data and knowledge gaps for the species of *Oligoneuriopsis*.

**Species**	**Imago described**	**Nymph described**	**COI sequenced**
*O. dobbsi*	+	+	+
*O. elisabethae*		+	
*O. jessicae*		+	
*O. lawrencei*	+	+	+
*O. orontensis*	+	+	+
*O. skhounate*	+	+	+
*O. villosus*		+	+
*O.* sp. (Iran)	+		

**Figure 16.**
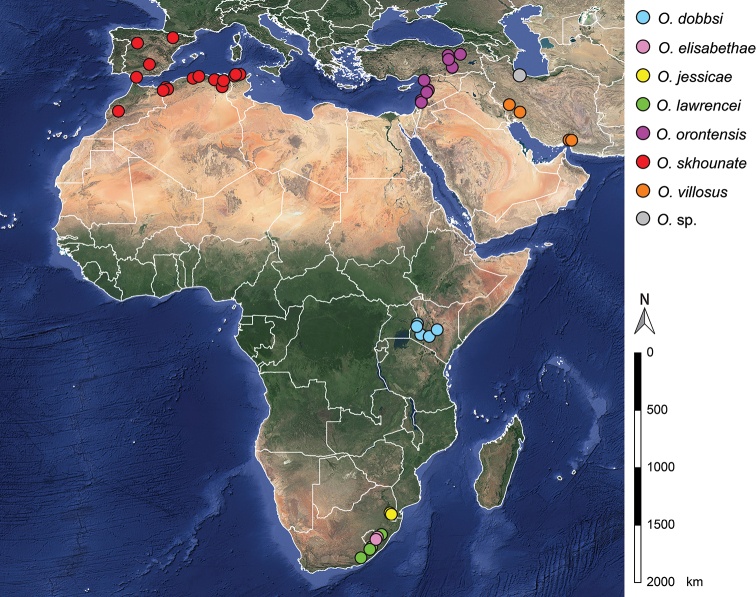
Map with indication of all records of the eight *Oligoneuriopsis* species.

We reconstructed the known distribution of the genus based on the material examined in this publication, and information from other sources (Eaton 1912; Koch 1980, 1988; Salur et al. 2016; Sroka et al. 2019; Bouhala et al. 2020; Yanai et al. 2020). The genus seems to be distributed in a narrow line up continental (Fig. 16), following the escarpment associated with the Great Rift Valley (Jordan Rift, Red Sea Rift, and East African Rift). In the north, this distribution branches eastwards (Iran) and westwards (North Africa and Iberian Peninsula).

With a cluster of species recorded in South Africa, the distribution suggests a once more widely spread group that has since been restricted to cooler climate at higher altitude as Gondwanaland moved north into warmer climatic zones, isolating populations of this originally cool-adapted lineage to produce the species we see today. Massariol et al. (2019), in a global study encompassing all Oligoneuriidae genera, deduced through molecular phylogeny dating that the family had a Gondwana origin, some 150 Mya, with the lineage producing *Oligoneuriopsis* and *Oligoneuriella*, the Oligoneuriellini, arising during the early Eocene, around 50 Mya. This fits the geological history of the Great Rift Valley: Roberts et al. (2012) indicate that uplift of the shoulders of the rift valley began during the early Eocene, some 45–40 Mya, which ties in with the estimate date of the diverging of the lineage. A possibly South African origin for *Oligoneuriopsis* can be hypothesized, with subsequent colonisation northwards through Kenya (*O. dobbsi*), then North Africa and the Iberian Peninsula (*O. skhounate*) or the Levant with an extension to the Middle East (*O. orontensis*, *O. villosus*). *Oligoneuriopsis lawrencei* being a sister-species to all other species, followed by *O. dobbsi* as a sister-species to the remaining ones (Fig. 1), support this hypothesis. A dated molecular phylogeny, involving some more conservative markers such as nuclear ones, for all *Oligoneuriopsis* species would be helpful to elucidate this. However, this is currently not possible as fresh material for most taxa is scarce.

## Supplementary Material

XML Treatment for Oligoneuriopsis lawrencei

XML Treatment for Oligoneuriopsis dobbsi

XML Treatment for Oligoneuriopsis jessicae

XML Treatment for Oligoneuriopsis elisabethae

XML Treatment for Oligoneuriopsis skhounate

XML Treatment for Oligoneuriopsis orontensis

XML Treatment for Oligoneuriopsis villosus

XML Treatment for Oligoneuriopsis
